# Simultaneous Expression of Long Non-Coding RNA *FAL1* and Extracellular Matrix Protein 1 Defines Tumour Behaviour in Young Patients with Papillary Thyroid Cancer

**DOI:** 10.3390/cancers13133223

**Published:** 2021-06-28

**Authors:** Seonhyang Jeong, Seul-Gi Lee, Hyunji Kim, Gibbeum Lee, Sunmi Park, In-Kyu Kim, Jandee Lee, Young-Suk Jo

**Affiliations:** 1 Department of Internal Medicine, Yonsei University College of Medicine, 50-1 Yonsei-ro, Seodaemun-gu, Seoul 03722, Korea; BAMBI_89@yuhs.ac (S.J.); SUNMIP@yuhs.ac (S.P.); 2 Department of Surgery, Eulji University School of Medicine, 95 Dunsanseo-ro, Seo-gu, Daejeon 35233, Korea; rtigger@naver.com; 3 Yonsei Cancer Center, Open NBI Convergence Technology Research Laboratory, Severance Hospital, Department of Surgery, Yonsei University College of Medicine, 50-1 Yonsei-ro, Seodaemun-gu, Seoul 03722, Korea; HJKIM0612@yuhs.ac (H.K.); GBLEE@yuhs.ac (G.L.); IGKIM@yuhs.ac (I.-K.K.)

**Keywords:** *FAL1*, *ECM1*, thyroid neoplasm, prognosis, *RAS*, immunology

## Abstract

**Simple Summary:**

*FAL1* upregulation has been reported in many types of human cancers. The up-regulatory mechanism was identified in ovarian cancer but was not investigated in other type of cancers. Using The Cancer Genome Atlas (TCGA) database, we identified simultaneous upregulation of *FAL1* adjacent to chromosome 1q21.3. Among 53 putative transcription factors for *FAL1* and neighbouring genes, we selected c-JUN and JUND as the best candidates. This simultaneous upregulation defines molecular biological features representing RAS-driven PTC-enriched immune-related gene sets. These findings suggest that the simultaneous upregulation might be a potential diagnostic and therapeutic target for RAS-driven PTC.

**Abstract:**

We investigated the regulatory mechanism of *FAL1* and unravelled the molecular biological features of *FAL1* upregulation in papillary thyroid cancer (PTC). Correlation analyses of *FAL1* and neighbouring genes adjacent to chromosome 1q21.3 were performed. Focal amplification was performed using data from copy number alterations in The Cancer Genome Atlas (TCGA) database. To identify putative transcriptional factors, PROMO and the Encyclopaedia of DNA Elements (ENCODE) were used. To validate c-JUN and JUND as master transcription factors for *FAL1* and *ECM1*, gene set enrichment analysis was performed according to *FAL1* and *ECM1* expression. Statistical analyses of the molecular biological features of *FAL1-* and *ECM1-*upregulated PTCs were conducted. *FAL1* expression significantly correlated with that of neighbouring genes. Focal amplification of chromosome 1q21.3 was observed in ovarian cancer but not in thyroid carcinoma. However, PROMO suggested 53 transcription factors as putative common transcriptional factors for *FAL1* and *ECM1* simultaneously. Among them, we selected c-JUN and JUND as the best candidates based on ENCODE results. The expression of target genes of JUND simultaneously increased in *FAL1-* and *ECM1-*upregulated PTCs, especially in young patients. The molecular biological features represented RAS-driven PTC and simultaneously enriched immune-related gene sets. *FAL1* and *ECM1* expression frequently increased simultaneously and could be operated by JUND. The simultaneous upregulation might be a potential diagnostic and therapeutic target for RAS-driven PTC.

## 1. Introduction

Papillary thyroid cancer (PTC) is the most common endocrine malignancy, and its incidence has been increasing worldwide [1,2]. The main reason for this increase has been attributed to the widespread application of highly sensitive ultrasound to health screenings [3]. In this context, a new approach called active surveillance is being widely studied in the clinical setting [4]. However, basic experiments and clinical studies on thyroid cancer occurring in young patients and refractory thyroid cancer relatively commonly observed in the elderly are also being conducted [5,6,7,8]. These efforts have led to the development of various tyrosine kinase inhibitors, including targeted therapy for *BRAFV600E*, for the clinical setting. However, owing to limited therapeutic efficacy, there remains a need for new therapeutic agents. Unfortunately, no new emerging therapeutic targets have been suggested [9].

Non-coding RNA (ncRNA), a molecule that is not translated into protein, is present abundantly and performs important biological functions [10,11,12]. Among various ncRNAs such as small nucleolar RNAs (snoRNAs), microRNAs (miRNAs), small interfering RNA (siRNA), small nuclear RNA (snRNA), extracellular RNA (exRNA), piwi-interacting RNA (piRNAs), and long ncRNAs (lncRNAs) [13,14], previous studies have shown that lncRNAs act as tumour susceptible genes in PTC. For example, low expression of PTC susceptibility candidate 2 (*PTSC2*) was observed in PTC tumours, which, in turn, affected the expression of genes related to cell cycle and cancer [15]. The polymorphism rs944289 on PTC susceptibility candidate 3 (*PTSC3*) was reported to predispose to PTC [16]. BRAF-activated lncRNA (*BANCR*) is upregulated in PTC, thereby promoting cell proliferation through autophagy regulation [17].

Recently, our group reported that the oncogenic activity of *f*ocally amplified lncRNA on chromosome 1 (*FAL1*, *ENSG00000228126*) is attributed to the expression of genes related to the cell cycle, including transcription factor E2F transcription factor 1 (*E2F1*), E2F transcription factor 2 (*E2F2*), and cyclin D1 in thyroid cancers [18]. In this study, we investigated the mechanism underlying *FAL1* upregulation in PTC and found that extracellular matrix protein 1 (*ECM1*) expression was simultaneously upregulated with *FAL1* expression. In addition, we explored the biological and clinical functions of *FAL1* and *ECM1* and demonstrated the significance of simultaneous expression of *FAL1* and *ECM1*.

## 2. Materials and Methods

### 2.1. Analysis of lncRNA and mRNA Expression Using Public Databases

lncRNA expression values were collected using The Atlas of Noncoding RNAs in Cancer (TANRIC) derived from The Cancer Genome Atlas (TCGA) RNA-seq database [19]. TANRIC provided the quantified expression level of lncRNA as read per kilobase million (RPKM) based on the Binary Alignment/Map format File (BAM file, *.bam). Using these datasets, the expression level of the lncRNA *FAL1* was confirmed in 20 types of human cancer: colon adenocarcinoma (COAD; 157 cases), rectum adenocarcinoma (READ; 71 cases), uterine corpus endometrioid carcinoma (UCEC; 316 cases), kidney chromophobe (KICH; 66 cases), ovary serous cystadenocarcinoma (OV; 412 cases), liver hepatocellular carcinoma (LIHC; 200 cases), prostate adenocarcinoma (PRAD; 374 cases), kidney renal clear cell carcinoma (KIRC; 448 cases), stomach adenocarcinoma (STAD; 285 cases), bladder urothelial carcinoma (BLCA; 252 cases), lung squamous cell carcinoma (LUSC; 220 cases), kidney renal papillary cell carcinoma (KIRP; 198 cases), cervical squamous cell carcinoma and endocervical adenocarcinoma (CESC; 196 cases), lung adenocarcinoma (LUAD; 488 cases), skin cutaneous melanoma (SKCM; 226 cases), breast invasive carcinoma (BRCA; 837 cases), head and neck squamous cell carcinoma (HNSC; 426 cases), glioblastoma multiforme (GBM; 154 cases), thyroid carcinoma (THCA; 497 cases), and brain low grade glioma (LGG; 486 case). To compare the expression values of *ECM1* and *ADAMTSL4* between normal tissues and PTC, mRNA and lncRNA expression values for the normal tissue cohort were collected from the TCGA THCA RNA-seq database and TANRIC, respectively. We also selected GSE127083 from the GEO (Gene Expression Omnibus) database as in vitro validation set in addition to TCGA data.

### 2.2. Analysis of Copy Number Alteration

To determine copy number alterations in TCGA ovarian cancer (OVCA) and TCGA THCA, Genomic Identification of Significant Targets in Cancer (GISTIC) data from the Broad Firehose infrastructure were used [20]. GISTIC statistically calculated the copy number alteration occurring in many patient specimens. The data were sorted based on the genomic build hg19. The threshold used for DNA copy number amplification was 0.1, the confidence level was 0.99, and the *q*-value cut-off was 0.25.

### 2.3. Prediction and Validation of Putative Transcription Factors

The binding site and transcription factors of *FAL1* and *ECM1* were predicted using PROMO and the Encyclopaedia of DNA Elements (ENCODE), respectively. PROMO, which used a transcription factor source from the TRANSFAC^®^ database, predicted potential transcription factor-binding sites (TFBS). The upstream 1 kb sequence of *FAL1* and *ECM1* was input, and the dissimilarity rate was analysed using the default value (15%; 85% similarity). Information related to gene regulation based on ENCODE data was verified using the Ensembl Genome Browser. The regulatory elements of each gene were identified using chromatin immunoprecipitation followed by sequencing (ChIP-Seq) data from ENCODE, and each motif score was selected by applying a *p*-value threshold of 0.01. Gene set enrichment analysis (GSEA) was performed using software, version 4.1.0, combined with Gene Ontology [21]. The normalised enrichment score (NES) calculated by GSEA described the correlation between the gene set and the expression dataset. Permutations were performed 1000 times according to the basic weighted enrichment statistic, and genes were ranked according to the level of differential expression between the two groups. We selected a set of important genes based on a *p*-value of ≤0.05 and a false discovery rate (FDR) *q*-value of <0.25.

### 2.4. Statistical Analysis

Statistical analysis was performed using Prism (GraphPad Software, San Diego, CA, USA) or SPSS, version 25.0, for Windows (IBM Corp., Armonk, NY, USA). Data are presented as mean ± SD. Statistical comparisons of continuous variables were performed using Student’s t-test or analysis of variance, and group comparisons were performed using χ^2^ test or linear association. Gene expression associations were investigated using the Pearson correlation coefficients.

## 3. Results

### 3.1. Positive Correlation between FAL1 and ECM1 Expression in Human Cancers

**Figure 1:**
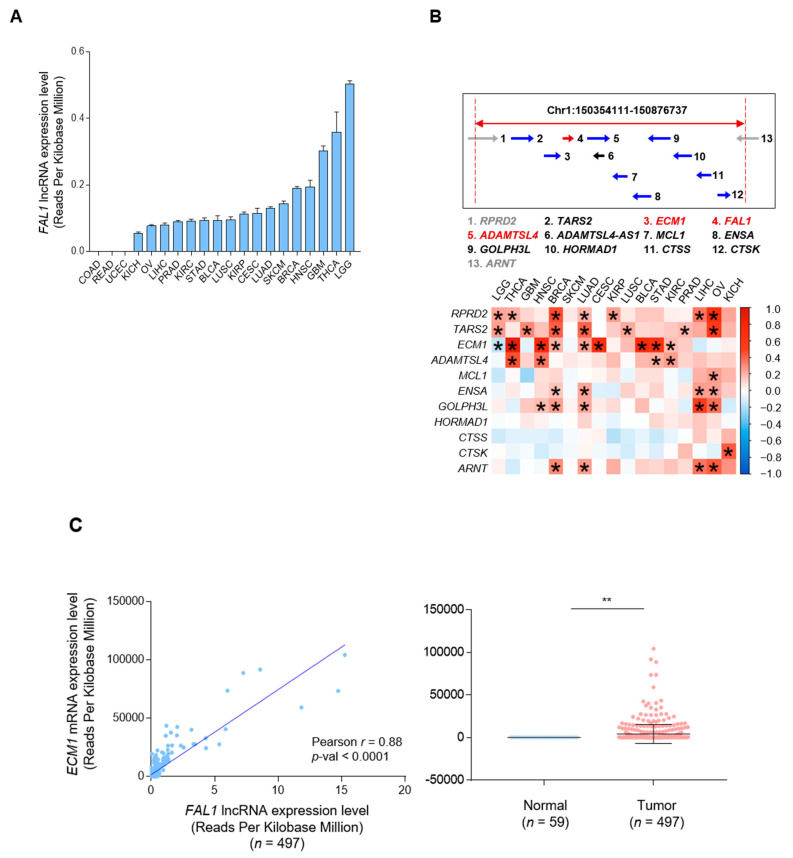

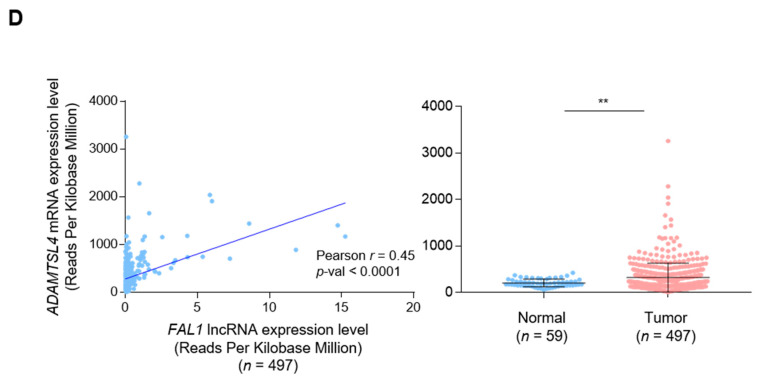
(**A**) Positive correlation of *FAL1* expression with the expression of neighbouring genes adjacently located on chromosome 1q21.3. A. *FAL1* expression status of 20 types of human cancers. (**B**) Location of *FAL1* and neighbouring genes on chromosome 1q21.3 and correlation status of *FAL1* with neighbouring genes in terms of expression in 17 human cancers. (**C**) Positive correlation of *FAL1* expression and *ECM1* expression, and upregulated expression of *ECM1* in tumour samples compared to that in normal tissues in THCA. (**D**) Positive correlation of *FAL1* expression with *ADAMTSL4* expression and upregulated expression of *ADAMTSL4* in tumour samples compared to that in normal tissues in THCA. Abbreviations: *FAL1*, focally amplified lncRNA on chromosome 1; RPRD2, regulation of nuclear pre-MRNA domain-containing 2; TARS2, threonyl-tRNA synthetase 2, mitochondrial; *ECM1*, extracellular matrix protein 1; *ADAMTSL4*-AS1, *ADAMTSL4* antisense RNA 1; MCL1, myeloid cell leukaemia 1; ENSA, endosulfine alpha; GOLPH3L, Golgi phosphoprotein 3-like; HORMAD1, HORMA domain-containing 1; CTSS, cathepsin S; CTSK, cathepsin K; ARNT, aryl hydrocarbon receptor nuclear translocator. The arrows indicate the direction of transcription. Average values were compared using an unpaired t-test. In the scatter plots, data are expressed as the mean ± SD. Correlation coefficient: Pearson’s r. * *p* < 0.05, ** *p* < 0.01. All *p*-values are two-sided.

Upregulation of *FAL1* expression was first reported in ovarian cancer. Focal amplification of chromosome 1q21.3 is the mechanism underlying the upregulated expression of *FAL1* [22]. To validate this upregulatory mechanism in PTC, we first investigated the expression status of *FAL1* using pan-cancer data from TCGA. Among 20 types of human cancers, COAD, READ, and UCEC did not show any expression of *FAL1*. However, brain LGG, THCA, and GBM presented higher expression values than the other types of human cancers (Figure 1A). Interestingly, the expression of *FAL1* in OVCA was not higher than that in other types of human cancers. Because the upregulated expression of genes by focal amplification is inevitably accompanied by increased expression of genes present at the same location on the chromosome, we identified the genes located around *FAL1* (Figure 1B). *ECM1* and *ADAMTS* (disintegrin and metalloproteinase with thrombospondin motifs)-like 4 (*ADAMTSL4*) were located before and after *FAL1* on Chr1:150354111-150876737. An analysis of the correlation between the expression of *FAL1* and that of the neighbouring genes was performed, and the results showed that the expression of threonyl-tRNA synthetase 2, mitochondrial (*TARS2*), *ECM1*, *ADAMTSL4*, etc., in most tumours wherein *FAL1* expression was observed was positively correlated with *FAL1* expression (Figure 1B). Whereas the correlation of *FAL1* with *RPRD2* and *TARS2* did not show strong positive correlation coefficients in TCGA THCA (Figure S1), as shown in Figure 1C,D, the expression of *ECM1* and *ADAMTSL4* showed a positive correlation with *FAL1* expression and was associated with upregulated expression of *FAL1* in PTC compared to that in the normal thyroid tissues. Taken together, we postulated that *FAL1* expression was coupled with the upregulated expression of neighbouring genes such as *ECM1* and *ADAMTSL4*.

### 3.2. Focal Amplification of the FAL1 Gene in Ovarian Cancer but Not In PTC

**Figure 2:**
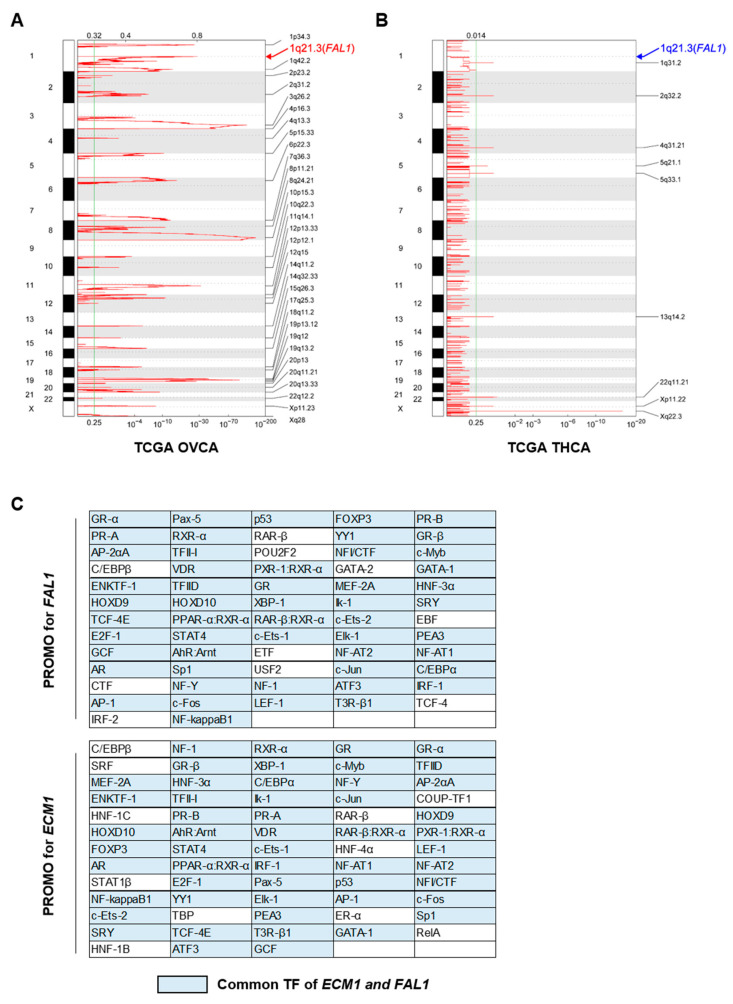
Analysis of focal amplification using data of copy number alterations from TCGA OVCA and TCGA THCA. (**A**) Results of analysis of the focal amplification in the whole chromosome from TCGA OVCA. (**B**) Results of analysis of the focal amplification in the whole chromosome from TCGA THCA. (**C**)Results from PROMO analysis to identify putative TFBS in DNA sequences of the *FAL1* and *ECM1* genes. Abbreviations: TF, transcription factor; TFBS, transcription factor-binding sites; TCGA, The Cancer Genome Atlas; THCA, thyroid carcinoma; OVCA, ovarian cancer. The arrows indicate chromosome 1q21.3, which was reported as a focal amplification lesion for *FAL1* upregulation in OVCA.

Our analysis for the expression of *FAL1* and neighbouring genes suggested simultaneous upregulated expression of those genes, and we investigated the amplification status of genes based on data of copy number alterations (CNAs) from TCGA OVCA and TCGA THCA. As previously reported, focal amplification of 1q21.3 was clearly observed in OVCA (Figure 2A), but no amplification signal at the same position was observed in THCA (Figure 2B), suggesting that simultaneous upregulated expression of *FAL1* and the neighbouring genes was generated by different mechanisms from focal amplification in PTC. Supporting our idea, the comparison of 1q arm-level amplification in TCGA THCA according to the *FAL1* expression status did not show any significant difference (Table S1). Recent efforts to understand the regulatory mechanism of lncRNA expression have emphasised the importance of transcription factors in the expression of other coding genes. In line with this idea, we decided to evaluate the effective transcription factors for the promoter areas of *FAL1* and *ECM1*. To achieve our goal, we identified putative TFBS in DNA sequences defined in TRANSFAC, using PROMO [23,24]. This virtual laboratory suggested 62 transcription factors for *FAL1* and 63 transcription factors for *ECM1* (Figure 2C). As expected, most putative transcription factors overlapped in both *FAL1* and *ECM1* (*n* = 53). Taken together, the simultaneous expression of *FAL1* and *ECM1* might be generated by common transcription factors rather than by gene amplification.

### 3.3. JUND as a Candidate for Common Transcription Factor for FAL1 and ECM1

**Figure 3:**
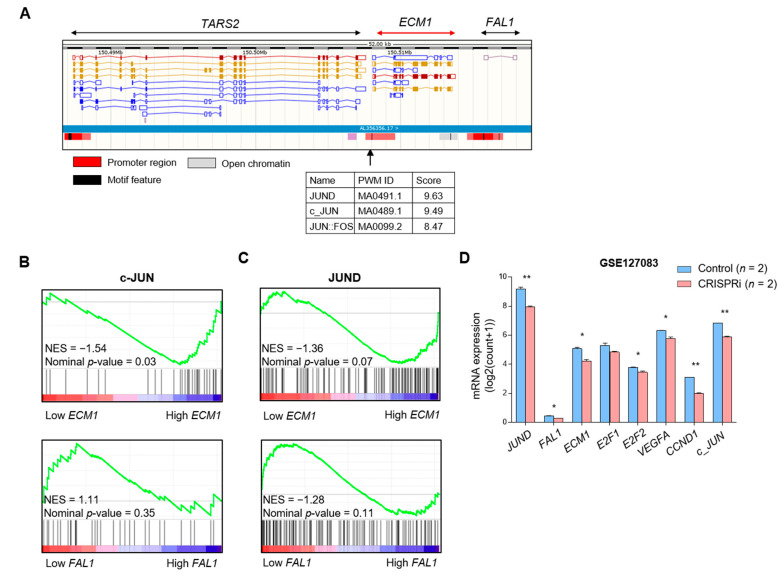

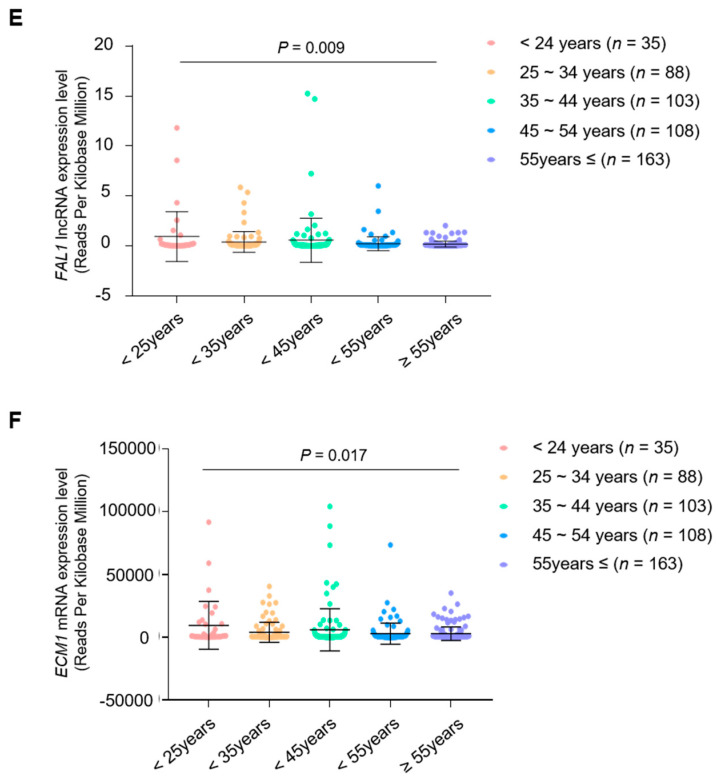
Results of the analysis using the Encyclopaedia of DNA Elements (ENCODE) and *FAL1* and *ECM1* expression according to patients’ age. (**A**) The TFBS of JUND, c-JUN, and JUN-FOS in the promoter area of *ECM1* and *FAL1*. (**B**) Expression of the transcriptional target genes of c-JUN according to *ECM1* and *FAL1* expression status in TCGA THCA. (**C**) Expression of the transcriptional target genes of JUND according to *ECM1* and *FAL1* expression status in TCGA THCA. (**D**) Selected target gene expressions were identified in GSE127083 RNA-seq dataset for K562 cells treated with *JUND*-targeted CRISPRi. (**E**) *FAL1* expression status according to patients’ age (**F**) *ECM1* expression status according to patients’ age. Abbreviations: JUND, junD proto-oncogene; AP-1, transcription factor subunit; JUN, jun proto-oncogene; FOS, fos proto-oncogene; TFBS, transcription factor-binding sites; TCGA, The Cancer Genome Atlas; THCA, thyroid carcinoma; E2F1, transcription factor E2F transcription factor 1; E2F2, transcription factor E2F transcription factor 2; VEGFA, vascular endothelial growth factor A; CCND1 Cyclin D1. Average values were compared using ANOVA, Student’s *t*-test. In the scatter plots, data are expressed as mean ± SD. All *p*-values are two-sided. * *p* < 0.01, ** *p* < 0.001.

To select the most reliable transcription factor from TFBS, we examined the location of TFBS on the chromosome in detail using the Encyclopaedia of DNA Elements (ENCODE), which identifies functional elements in the human genomes [25]. In the case of *RPRD2* and *TARS*, PROMO suggested 66 transcription factors for *RPRD2* and 70 transcription factors for *TARS2*. However, unfortunately, ENCODE did not have TFBS for *RPRD2* (Figure S2). Interestingly, we observed that the TFBS of c-JUN and JUND was present in the promoter of the *ECM1* gene, and the TFBS for *FOS*, a binding partner, was also present (Figure 3A). Based on this finding, we hypothesised that the expression of JUN target genes might be increased in *FAL1-* and *ECM1-*upregulated PTC, if the expression of *FAL1* and *ECM1* was increased by JUN transactivation. To validate our hypothesis, we performed GSEA by dividing the TCGA THCA into two groups according to the *FAL1* and *ECM1* expression status. The target genes of c-JUN were co-ordinately enriched in the high *ECM1* group but not in the high *FAL1* group (Figure 3B). However, the target genes for JUND were co-ordinately enriched in the high *ECM1* group and showed a tendency of upregulated expression in the high *FAL1* group (Figure 3C) even though *JUND* expression was not correlated with *FAL1* and *ECM1* expression (Figure S3). In addition, we confirmed that the expression of the *FAL1*, *ECM1*, and *FAL1* target genes was also decreased by CRISPRi *JUND* in leukemia cells (Figure 3D). Although we did not verify these results from a virtual laboratory through a wet lab-based approach, we thought that these data fully supported our idea that simultaneous expression of *FAL1* and *ECM1* was generated, not by focal amplification but by common transcription factors such as JUND.

*FAL1* was first investigated in ovarian high-grade serous carcinoma (HGSC), showing a different correlation pattern compared to TCGA THCA (Figure 1B). We thought this difference might indicate a different regulatory mechanism of *FAL1* upregulation according to cancer types. As described, in HGSC, focal amplification increased *FAL1* upregulation. In the case of GBM, the regulatory mechanism was not investigated. To understand the different regulatory mechansim in HGSC and GBM, we performed GSEA using c-JUN and JUND target genes in HGSC and GBM. High *ECM1* expression was related to coordinately enrichment of c-JUN and JUND target genes but high *FAL1* expression was not (Figure S4). This data indicated that JUND was not involved in simutaneous up regulation of *ECM1* and *FAL1* in HGSC and GBM.

### 3.4. Clinical Relevance of the Simultaneous Expression of FAL1 and ECM1

**Figure 4:**
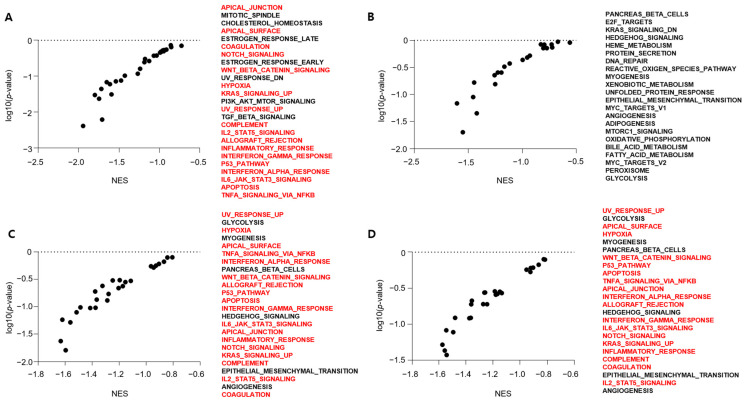
Results of GSEA according to patients’ age, *FAL1* upregulation and *ECM1* upregulation. (**A**)Representative gene sets co-ordinately enriched in the young patient group. (**B**) Representative gene sets co-ordinately enriched in the old-aged group. (**C**) Representative gene sets co-ordinately enriched in the *FAL1*-upregulated group. (**D**) Representative gene sets co-ordinately enriched in the *ECM1*-upregulated group. Red colour refers to gene sets overlapping in the young-aged group and the *FAL1*- and *ECM1*-upregulated group. Abbreviations: GSEA, gene set enrichment analysis; NES, normalised enrichment score.

To determine the clinical implication of the simultaneous expression of *FAL1* and *ECM1*, we analysed the clinicopathological features of high *FAL1* and *ECM1* expression groups. Among the results, the most interesting finding was that *FAL1* and *ECM1* expression was upregulated in young patients aged <45 years (Figure 3D,E). In line with this finding, we performed GSEA to understand the differences in gene expression patterns according to age (young vs. old age) and found that KRAS- and immune-related gene sets were co-ordinately enriched in the young age group (<45 years old), whereas metabolism- and epithelial–mesenchymal transition-related gene sets were co-ordinately enriched in the old age group (≥55 years old) (Figure 4A,B). According to high *FAL1* or *ECM1* expression, KRAS- and immune-related gene sets were co-ordinately enriched (Figure 4C,D). Taken together, *FAL1* and *ECM1* upregulated expression was frequently observed in the young patient group, and this upregulation might contribute to defining tumour behaviour in the young patient group. To validate our hypothesis that simultaneous expression of *FAL1* and *ECM1* might be related to RAS-driven PTC, we compared the molecular biological features of PTC according to *FAL1* and *ECM1* expression (Table 1 and Table 2). *FAL1*-upregulated PTC presented fewer mRNA clusters but more miRNA clusters. RAS mutations were frequently detected in *FAL1*-upregulated PTC, whereas the frequency of BRAF mutations was relatively low. According to the frequent RAS mutation in *FAL1*-upregulated PTC, the RAS/RAF, ERK, and differentiation scores were compatible with RAS-driven PTC. However, serine 473 phosphorylation of PKB/AKT increased in *FAL1*-upregulated PTC. In the analysis of *ECM1*-upregulated PTC, frequent RAS mutations and a relatively low frequency of BRAF mutations were also observed as *FAL1*-upregulated PTC. However, the frequency of TERT promoter mutation increased, and the ERK score also increased compared to that in *ECM1*-downregulated PTC. In summary, all these molecular biological features might be related to the aggressive behaviour of RAS-driven tumours.

## 4. Discussion

The incidence of PTC has been increasing since the last two decades in Korea even though there is controversy regarding the overtreatment of papillary thyroid microcarcinoma [3,26]. Recent clinical evidence has suggested the optimal indication of active surveillance for this indolent carcinoma [27]. Refractory thyroid cancer is defined as a tumour with poor response to current treatments such as surgery and radioactive iodine therapy, and it could be managed with newly developed targeted therapy using sorafenib and vemurafenib. However, these novel therapeutics mainly target *BRAFV600E*-driven PTC. In fact, currently available drugs for *RAS*-driven PTCs are limited.

LncRNA has been investigated as a novel diagnostic and therapeutic target in many types of human cancers [12]. In the case of PTCs, lncRNAs were first reported as cancer susceptible genes such as *PTCSC1*, *PTCSC2,* and *PTCSC3*; following this, many interesting papers reported various kinds of tumour-suppressive or oncogenic lncRNAs in human PTC, such as Cancer Susceptibility 2 (*CASC2*), Promoter Of CDKN1A Antisense DNA Damage Activated RNA (*PANDAR*), Maternally Expressed 3 (*MEG3*), Non-Protein-Coding RNA, Associated With MAP Kinase Pathway And Growth Arrest (*NAMA*), HOX Antisense Intergenic RNA (*HOTAIR*), Nuclear Enriched Abundant Transcript 1 (*NEAT1*), Metastasis Associated Lung Adenocarcinoma Transcript 1 (*MALAT1*), Antisense Noncoding RNA in the INK4 Locus (ANRIL), Plasmacytoma Variant Translocation 1 (*PVT1*), and BANCR [28,29,30,31,32]. Our group also reported *LOC100507661* and *FAL1* as potential oncogenic lncRNAs in PTC [18,33]. In fact, *FAL1* was first investigated in ovarian high-grade serous carcinoma (HGSC). This interesting study suggested that the mechanism underlying the upregulated expression of *FAL1* was focal amplification of the chromosome-harbouring *FAL1* gene, as indicated by the name itself. *FAL1* expression was closely related to the upregulation of E2F1, suggesting the oncogenic function of the *FAL1*-promoting cell cycle [22]. First, we aimed to confirm the regulatory mechanism of *FAL1* in PTC by focal amplification. However, copy number alteration data from TCGA THCA did not show any amplification signals on chromosome 1q21.3, even though *FAL1* and the neighbouring genes were highly expressed in PTC with a highly positive correlation with each other. Based on this finding, we prepared a virtual laboratory for the identification of TFBS in *FAL1* and *ECM1* promoter sequences. Results from PROMO showed many putative transcription factors for both *FAL1* and *ECM1*. After the analysis using ENCODE, we selected c-JUN and JUND as the best candidates for simultaneous expression of *FAL1* and *ECM1*, supported by GSEA according to the *FAL1* and *ECM1* expression status. In fact, ENCODE was generated by CHIP-seq from 48 cell lines, indicating our analysis using ENCODE are based on bench work experiments. All these analyses suggested that *FAL1* upregulation in PTC might be directed by transcription factors such as JUND but not by focal amplification of *the FAL1*-located chromosome.

In terms of clinical and biological relevance, upregulated expression of *FAL1* and *ECM1* was seen in the young age group and *RAS*-like PTC. In fact, the old-aged group showed more aggressive clinical features. In addition, our biological understanding is that *BRAF*-like PTC is more aggressive, and currently developed novel therapeutics have focused on *BRAF*-like refractory PTC [5,17]. However, in clinical settings, we have not encountered young patients with highly aggressive clinical features and *RAS*-like PTC harbouring *TERT* promoter mutation. Interestingly, our GSEA according to *FAL1* and *ECM1* expression status showed that the upregulated expression of these genes was functionally related to *RAS* signalling and immune-related genes. In the past, we considered epithelial-to-mesenchymal transition (EMT) as a representative mechanism of carcinogenesis [4,6,34,35,36]. However, in recent years, the importance of factors determining the tumour microenvironment has not been revealed, and it is understood that the relationship between tumour cells and the surrounding cells, especially immunological interaction, is an important factor in determining the prognosis of a tumour. In line with the current advances in tumour immunology, our data may suggest that *RAS-*like PTC with *FAL1* and *ECM1* upregulation proceeds from carcinogenesis to aggressive PTC by an immunological mechanism compared to classical *BRAF*-like PTC.

Our data did not show any statistically significant difference in clinical features, except age. We believe that this is due to the characteristics of the TCGA THCA cohort, which makes it difficult to reflect the clinically significant role of *FAL1* and *ECM1* in a limited sample number of aggressive *RAS*-like PTC because the prognosis of *BRAF*-like PTC, which is more frequently observed, is generally poor. However, the coordinated enrichment of *RAS* signalling and immune-related genes was consistently observed in PTC harbouring upregulated expression of *FAL1* and *ECM1* and PTC in young patients; we believe that this finding will be an important reference and will serve as the basis for future studies.

## 5. Conclusions

In conclusion, our data suggest that *FAL1* upregulation might be induced by selective transcription factors such as JUND and could be a useful diagnostic and therapeutic marker in aggressive *RAS*-like PTC, especially in young patients. Further studies that include a large sample size should be conducted to confirm our data.

## Figures and Tables

**Table 1:** Molecular features of TCGA THCA according to *FAL1* expression status.

Molecular Feature	*FAL1*		*p* Value
	Low Expression *n* = 124 (%)	High Expression *n* = 124 (%)	
mRNA cluster number			
1	12 (9.8)	59 (48.8)	<0.0001 ^†^
2	13 (10.7)	22 (18.2)	
3	32 (26.2)	5 (4.1)	
4	39 (32.0)	8 (6.6)	
5	26 (21.3)	27 (22.3)	
miRNA cluster number			
1	2 (1.6)	2 (1.6)	<0.0001 ^†^
2	48 (39.0)	19 (15.4)	
3	26 (21.1)	7 (5.7)	
4	16 (13.0)	58 (47.2)	
5	16 (13.0)	19 (15.4)	
6	15 (12.2)	18 (14.6)	
RAS mutation			
Absent	122 (98.4)	89 (72.4)	<0.0001 ^†^
Present	2 (1.6)	34 (27.6)	
BRAF mutation			
Absent	31 (25.0)	97 (78.9)	<0.0001 ^†^
Present	93 (75.0)	26 (21.1)	
TERT promoter mutation			
Absent	88 (87.1)	81 (89.0)	0.688 ^†^
Present	13 (12.9)	10 (11.0)	
RAS/RAF score	−0.67 ± 0.49	0.21 ± 0.67	<0.0001 *
ERK score	11.37 ± 16.27	2.14 ± 20.26	0.001 *
Differentiation score	−0.49 ± 0.99	0.54 ± 1.08	<0.0001 *
Akt pT308	−0.02 ± 0.64	0.06 ± 0.47	0.275 *
Akt pS473	−0.02 ± 0.47	0.09 ± 0.34	0.047 *

TCGA, The Cancer Genome Atlas; THCA, thyroid carcinoma. ^†^ *p* values calculated by Student’s *t* test; * *p* values calculated by chi-square test or linear-by-linear association.

**Table 2:** Molecular features of TCGA THCA according to *ECM1* expression status.

Molecular Feature	*ECM1*		*p* Value
	Low Expression *n* = 124 (%)	High Expression *n* = 124 (%)	
mRNA cluster number			
1	58 (47.9)	51 (43.2)	0.190 ^†^
2	7 (5.8)	21 (17.8)	
3	36 (29.8)	5 (4.2)	
4	5 (4.1)	14 (11.9)	
5	15 (12.4)	27 (22.9)	
miRNA cluster number			
1	3 (2.5)	3 (2.4)	0.796 ^†^
2	16 (13.1)	22 (17.9)	
3	24 (19.7)	11 (8.9)	
4	46 (37.7)	51 (41.5)	
5	19 (15.6)	21 (17.1)	
6	14 (11.5)	15 (12.2)	
RAS mutation			
Absent	117 (95.1)	90 (73.2)	<0.0001 ^†^
Present	6 (4.9)	33 (26.8)	
BRAF mutation			
Absent	71 (57.7)	92 (74.8)	0.005 ^†^
Present	52 (42.3)	31 (25.2)	
TERT promoter mutation			
Absent	91 (95.8)	78 (84.8)	0.011 ^†^
Present	4 (4.2)	14 (15.2)	
RAS/RAF score	0.01 ± 0.81	0.07 ± 0.68	0.533 *
ERK score	−5.25 ± 23.26	4.92 ± 19.35	0.001 *
Differentiation score	0.32 ± 1.37	0.39 ± 1.00	0.674 *
Akt pT308	0.09 ± 0.54	0.06 ± 0.35	0.691 *
Akt pS473	0.14 ± 0.66	0.01 ± 0.46	0.122 *

TCGA, The Cancer Genome Atlas; THCA, thyroid carcinoma. ^†^ *p* values calculated by Student’s *t* test; * *p* values calculated by chi-square test or linear-by-linear association.

## Data Availability

A description of the publicly archived datasets analyzed in this study is detailed in the Section 2.

## Supplementary Materials

The following are available online at https://www.mdpi.com/article/10.3390/cancers13133223/s1, Figure S1. Correlation analysis of *FAL1* expression with *RPRD2* and *TARS2* expression in THCA. (A) Correlation of *FAL1* expression with *RPRD2* expression (*n* = 497). Figure S2. Confirmation of transcription factor binding site of *RPRD2* and *TARS2* promoters. (A) Results of PROMO analysis that predicted TFBS from the DNA sequence of *RPRD2* gene. Figure S3. Correlation analysis of *JUND* expression with *FAL1*, *ECM1* expression in THCA. (A) Correlation of *JUND* expression with *FAL1* expression (*n* = 497). Table S1: Comparison of 1q arm-level amplification in TCGA THCA according to *FAL1* expression status.
