# Supplementary material for: Simultaneous Expression of Long Non-Coding RNA FAL1 and Extracellular Matrix Protein 1 Defines Tumour Behaviour in Young Patients with Papillary Thyroid Cancer
Source: Cancers (Basel). 2021 Jun 28;13(13):3223. doi: 10.3390/cancers13133223 (PMC8268647; doi:10.3390/cancers13133223)
Supplement: Supplementary file 1 [file cancers-13-03223-s001.zip › cancers-1253443-supplementary.pdf]

cancers

Supplementary Materials

Simultaneous Expression of Long Non-coding RNA *FAL1* and Extracellular Matrix Protein 1 Defines Tumour Behaviour in Young Patients with Papillary Thyroid Cancer

Seonhyang Jeong, Seul-Gi Lee, Hyunji Kim, Gibbeum Lee, Sunmi Park, In-Kyu Kim, Jandee Lee * and Young-Suk Jo *

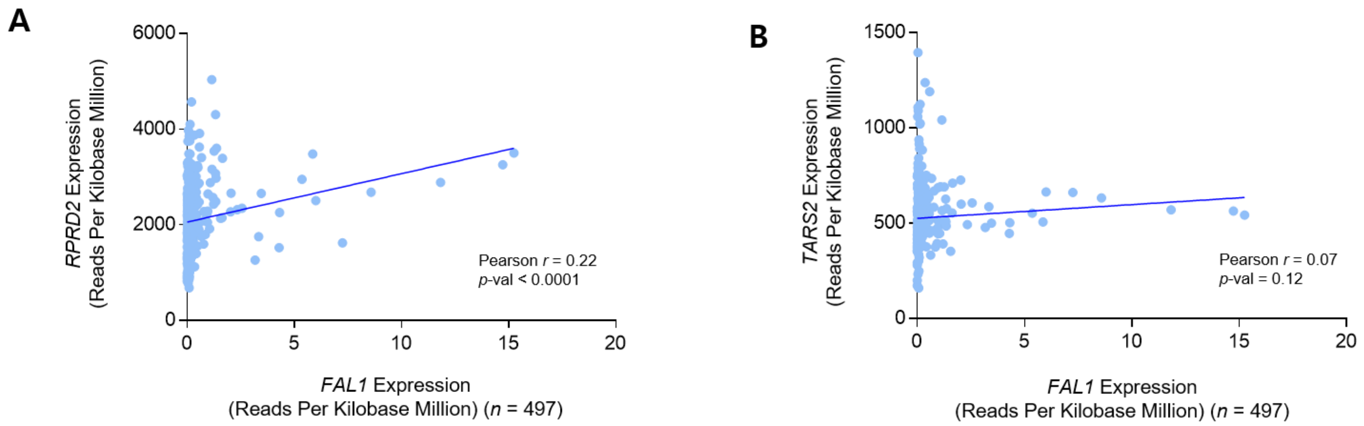

Figure S1. Correlation analysis of *FAL1* expression with *RPRD2* and *TARS2* expression in THCA. (**A**) Correlation of *FAL1* expression with *RPRD2* expression(n = 497). (**B**) Correlation of *FAL1* expression with *TARS2* expression(n = 497). Correlation analysis was performed using Pearson's correlation coefficient.

A

YY1	C/EBPβ	GR-α	NF-1	XBP-1
ENKTF-1	Pax-5	p53	HNF-1C	PR-β
PR-α	GR-β	VDR	FOXP3	PXR-1:RXR-α
RXR-α	NFI/CTF	GR	SRY	TCF-4E
RAR-β	T3R-β1	TFII-I	PEA3	MAZ
NF-AT2	STAT4	c-Ets-1	Elk-1	GATA-1
GCF	AP-2αA	MEF-2A	HNF-3α	ETF
C/EBPα	NF-Y	c-Jun	E2F-1	AhR:Arnt
c-Ets-2	LEF-1	TFIID	IRF-1	NF-AT1
c-Myb	HNF-1A	HNF-1B	TBP	STAT1β
RelA	AhR	WT1	E2F	POU2F2 (Oct-2.1)
ATF3	NF-kappaB	NF-kappaB1	Sp1	RBP-Jkappa
HOXD9	HOXD10	GATA-2	ER-α	EBF
PPAR-α:RXR-α	Ik-1			

PROMO for *RPRD2*

B

YY1	C/EBPβ	LEF-1	GR-α	AP-2αA
c-Jun	RAR-β:RXR-α	Pax-5	p53	XBP-1
C/EBPα	RXR-α	TFII-I	FOXP3	TFIID
HNF-3α	GR	GR-β	MEF-2A	HOXD9
HOXD10	c-Myb	AP-1	GATA-1	NFI/CTF
POU2F1	HNF-1C	HNF-1A	HNF-1B	POU2F2 (Oct-2.1)
PR-β	PR-α	NF-Y	NF-1	STAT4
c-Ets-1	ENKTF-1	E2F-1	Sp1	MAZ
WT1	ETF	NF-AT2	RelA	STAT1β
RBP-Jkappa	IRF-1	NF-AT1	Egr-3	USF2
T3R-β1	AR	SRY	TCF-4E	Elk-1
HNF-4α	ATF-1	c-Ets-2	PEA3	CTF
ER-α	NF-kappaB1	RAR-beta	TCF-4	VDR
PXR-1:RXR-α	NF-kappaB	GCF	ATF3	TBP

PROMO for *TARS2*

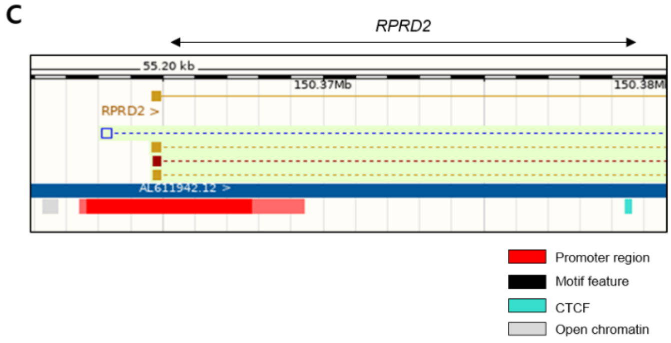

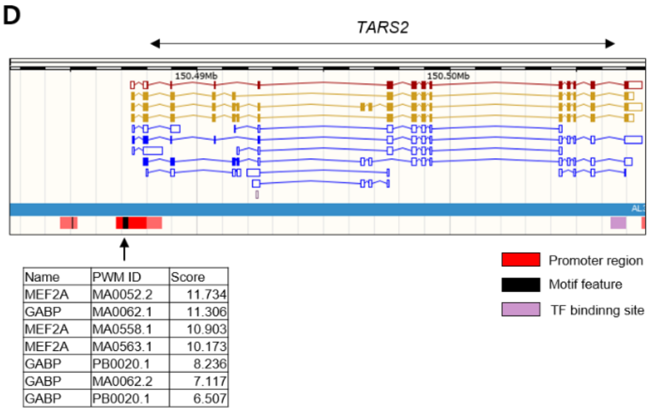

Name	PWM ID	Score
MEF2A	MA0052.2	11.734
GABP	MA0062.1	11.306
MEF2A	MA0558.1	10.903
MEF2A	MA0563.1	10.173
GABP	PB0020.1	8.236
GABP	MA0062.2	7.117
GABP	PB0020.1	6.507

Figure S2. Confirmation of transcription factor binding site of *RPRD2* and *TARS2* promoters. (**A**) Results of PROMO analysis that predicted TFBS from the DNA sequence of *RPRD2* gene. (**B**) Results of PROMO analysis that predicted TFBS from the DNA sequence of *TARS2* gene. (**C**) Results of analysis of the TFBS of *RPRD2* gene using ENCODE. (**D**) Results of analysis of the TFBS of *TARS2* gene using ENCODE.

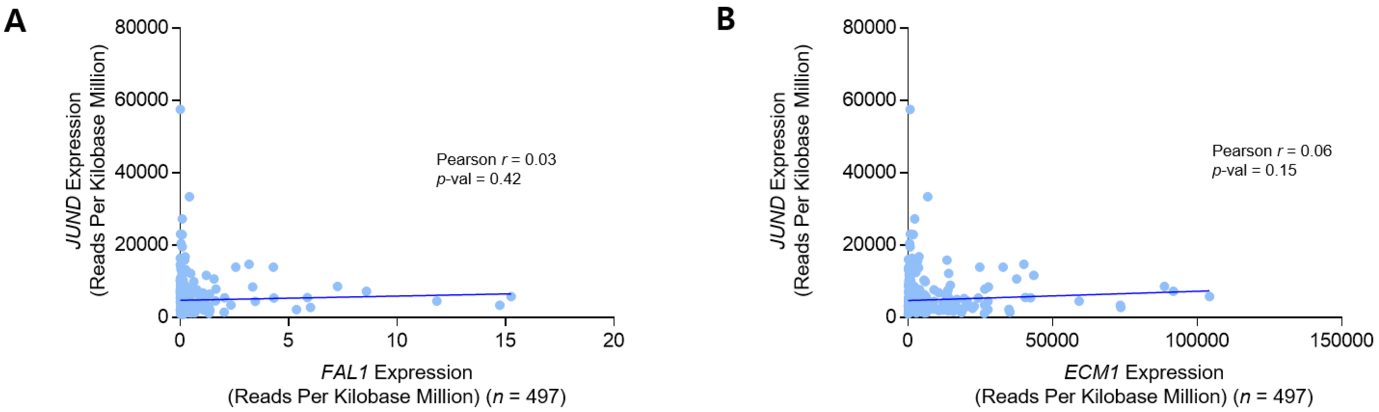

Figure S3. Correlation analysis of *JUND* expression with *FAL1*, *ECM1* expression in THCA. (**A**) Correlation of *JUND* expression with *FAL1* expression(n = 497). (**B**) Correlation of *JUND* expression with *ECM1* expression(n = 497). Correlation analysis was performed using Pearson's correlation coefficient.

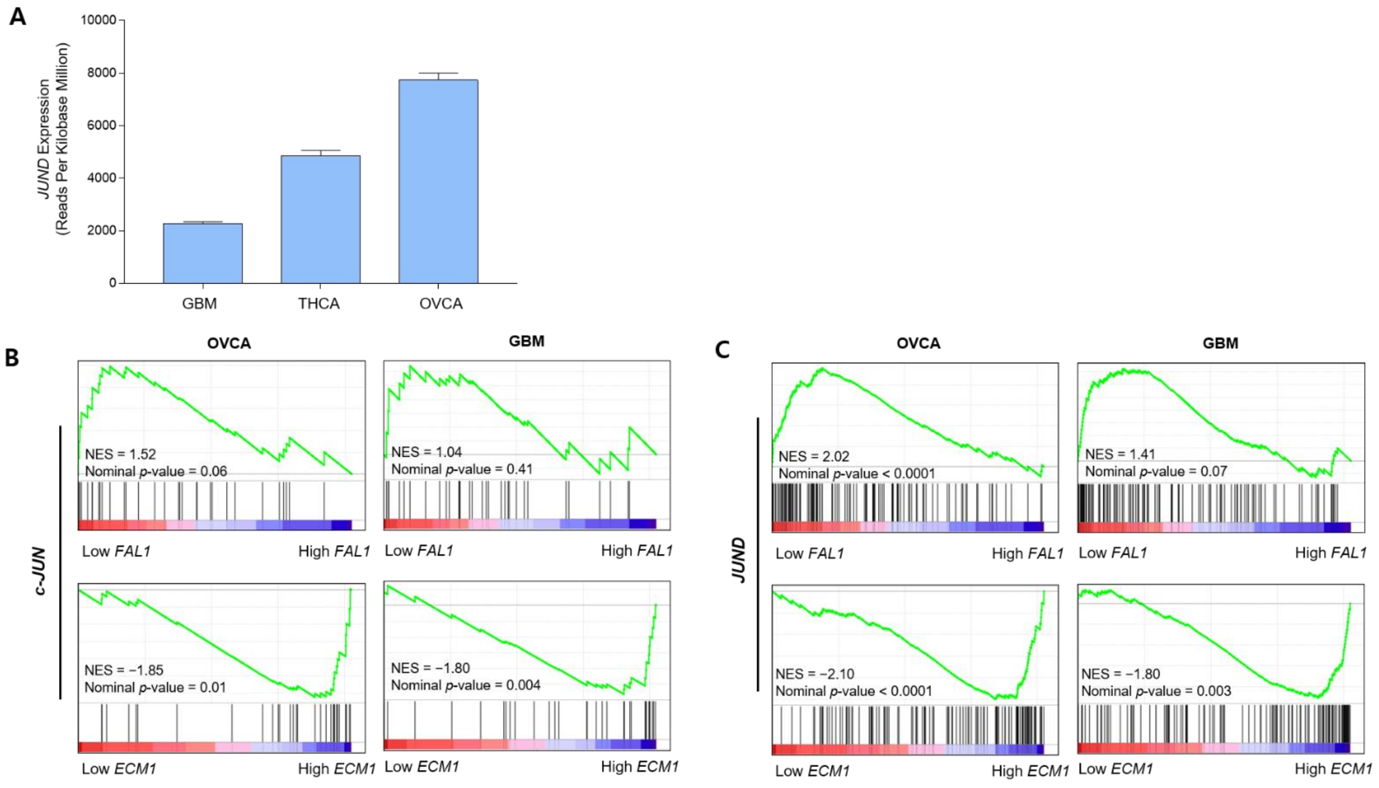

Figure S4. *JUND* expression and transcriptional target gene expression in GBM and OVCA. **A**.*JUND* expression status in GBM, THCA, and OVCA. **B**.Transcriptional target gene expression of *c-JUN* according to *ECM1* and *FAL1* expression patterns in GBM and OVCA. **C**.Transcriptional target gene expression of *JUND* according to *ECM1* and *FAL1* expression patterns in GBM and OVCA. Data are presented as mean±S.D. Abbreviations: GBE Glioblastoma, OVCA ovarian cancer.

Table S1. Comparison of 1q arm-level amplification in TCGA THCA according to *FAL1* expression status.

1q arm-level amplification	*FAL1* Expression		p value
	Low (n = 124, %)	High (n = 122, %)	
Absent	109 (87.9)	96 (78.7)	0.053
Present	15 (12.1)	26 (21.3)	
